# Proposal of Hard Palate-C2-Hyoid Bone Angle as a New Parameter Possibly Related to Dysphagia as a Postoperative Complication of Upper Cervical Fixation: A Case Series and Pilot Study

**DOI:** 10.7759/cureus.87564

**Published:** 2025-07-08

**Authors:** Yoshinori Maki, Toshinari Kawasaki, Tamaki Kobayashi, Yoshihiko Ioroi, Akio Goda, Motohiro Takayama

**Affiliations:** 1 Neurosurgery, Hikone Chuo Hospital, Hikone, JPN; 2 Spinal Surgery, Kyoto Katsura Hospital, Kyoto, JPN; 3 Department of Physical Therapy, Faculty of Health and Medical Sciences, Hokuriku University, Kanazawa, JPN

**Keywords:** complication, dysphagia, hyoid bone, occipitocervical fusion, upper cervical posterior fixation

## Abstract

Introduction: Occipitocervical fusion (OCF) and upper cervical posterior fixation can be performed to resolve occipitocervical or upper cervical instability. The cervical vertebral realignment following these procedures can result in dysphagia, which can decline patients’ postoperative status. Several radiological parameters on cervical lateral X-ray images were proposed to predict and avoid this postoperative dysphagia. However, previous parameters principally focus on cervical alignment and do not include the hyoid bone, which is an essential anatomical structure related to the swallowing mechanism.

Methods: In this case-series study, we enrolled a total of 14 patients (male: female = 10:4) with a mean age of 66.4 years who underwent OCF or upper cervical posterior fixation. We measured the following parameters on cervical lateral X-ray images: T1 slope, pharyngeal airway space, and O-C2, C1-C2, C2-C7, and pharyngeal tilt angles. In addition to these parameters, we also defined and measured a hard palate-C2-hyoid bone (H2H) angle, which reflects the anatomical relationship among the cervical vertebrae, palate, and hyoid bone. We compared pre- and postoperative changes of all the parameters between those patients with postoperative dysphagia after OCF and upper cervical posterior fixation and those without.

Results: Dysphagia occurred in two cases treated with OCF. A chronological change of the H2H angle in a case with postoperative dysphagia was plotted as an outlier on box-and-whisker plots, while any chronological changes of the other parameters in cases with dysphagia were not plotted as outliers.

Conclusion: The H2H angle could be related to the occurrence of postoperative dysphagia after OCF and upper cervical posterior fixation. This parameter should be evaluated with further studies.

## Introduction

Occipitocervical fusion (OCF) and upper cervical posterior fixation can be applied to resolve the instability of C1-C2 articulation resulting from various pathologies such as congenital malformations, infectious diseases, rheumatoid disease, tumors, and traumatic fractures [1-5]. These operations aim to achieve the realignment and stabilization of the targeted levels. The following perioperative complications were described: damage to spinal nerves and/or vertebral arteries, infection, cerebrospinal fluid leakage, trismus, respiratory distress, and dysphagia [1-5]. Dysphagia is a major clinical concern, as this complication can impede patients’ postoperative recovery. Although the occurrence of dysphagia in patients undergoing upper cervical posterior fixation seems rare compared to that in patients managed with OCF, postoperative dysphagia after upper cervical posterior fixation was also described in previous studies [3-5]. Moreover, the incidence of postoperative dysphagia after OCF remains high and can vary from 15.8% to 26.6% [6-11]. Prevention and prediction of this postoperative adverse event are quite important in clinical practice.

Radiological parameters on an X-ray lateral injection, including T1 slope, pharyngeal airway space (PAS), O-C2, C1-C2, C2-C7, and pharyngeal tilt angles, were proposed to prevent and predict dysphagia after OCF and upper cervical posterior fixation [1-6]. However, the previous parameters focus principally on the vertebral bone structure and do not include the hyoid bone, which is considered an essential anatomical structure related to the swallowing mechanism [12,13]. In a previous study by Badshah et al., the positions of the hyoid bone and hard palate related to cervical vertebral levels were evaluated on CT images [14]. However, any radiological parameter, including the plate and hyoid bone, which can be linked to dysphagia after OCF and upper cervical posterior fixation, is lacking in the literature. Therefore, we hypothesized that a radiological parameter inclusively containing the positions of the hyoid bone and hard palate could be clinically useful when the parameter is related to postoperative dysphagia following OCF and upper cervical posterior fixation.

This case-series study aimed to analyze radiological findings on X-rays before and after OCF and upper cervical posterior fixation, proposing a new useful parameter that might be related to postoperative dysphagia.

## Materials and methods

Selection of the cases in this study

**Figure 1:**
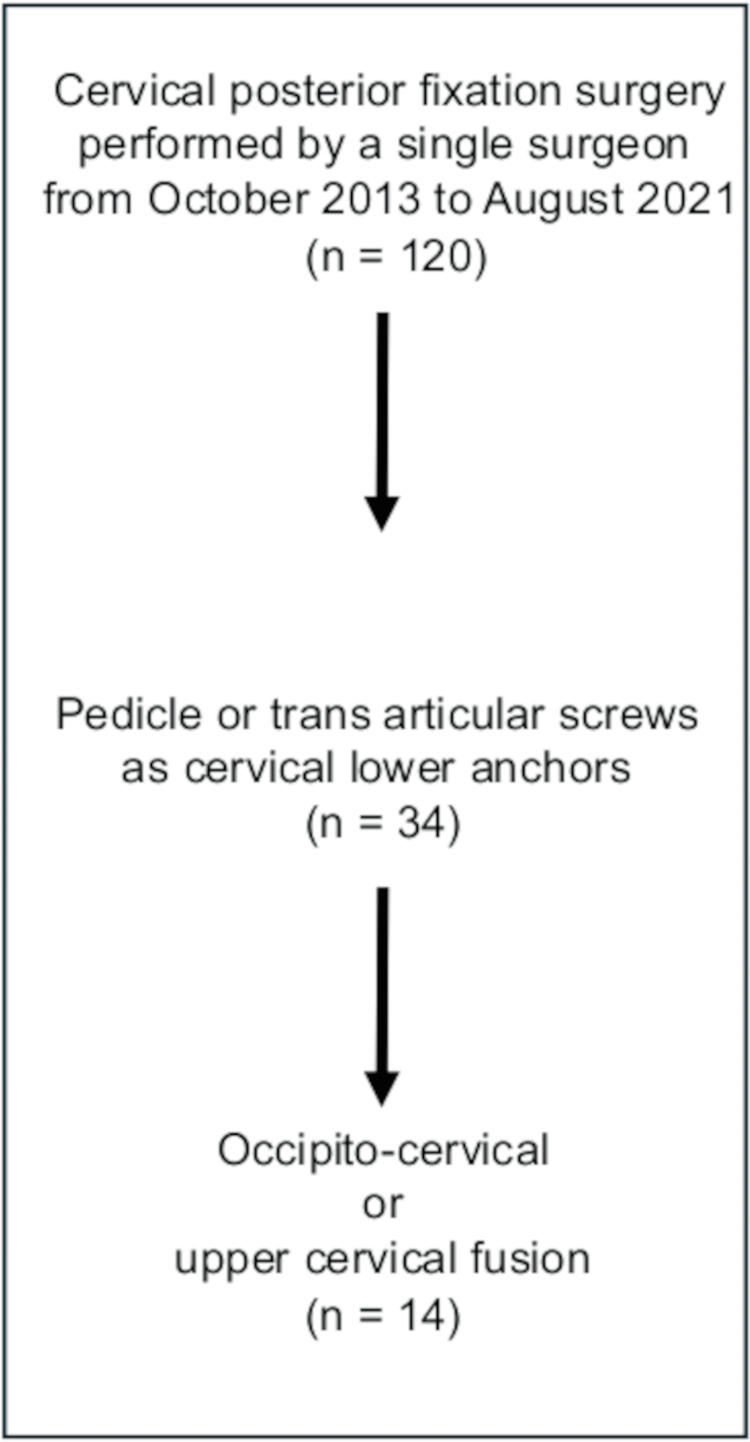
The flowchart of the enrolled cases in this study. Of the 120 patients who underwent cervical posterior fixation, 14 cases treated with occipito-cervical fusion or upper cervical fusion combined with pedicle screws and/or trans-articular screws of C1-C2 were enrolled in this study.

In this retrospective case series study, we analyzed the cases treated with cervical posterior fixation in a single institute from October 2013 to August 2021. Of all the 120 cases, we selected the cases in which posterior cervical fixation was performed with pedicle screws and/or transarticular screws of C1-C2. Among those 34 cases, we finally enrolled 14 cases treated with occipito-cervical fusion or upper cervical fusion (Figure 1). 

All the operations were performed by a single neurosurgeon (M.T.). Then, we divided the enrolled cases into two groups: a group in which postoperative dysphagia did not appear and the other in which postoperative dysphagia appeared. We measured the following radiological parameters on X-ray images [15].

Measured items on X-ray images 

**Figure 2:**
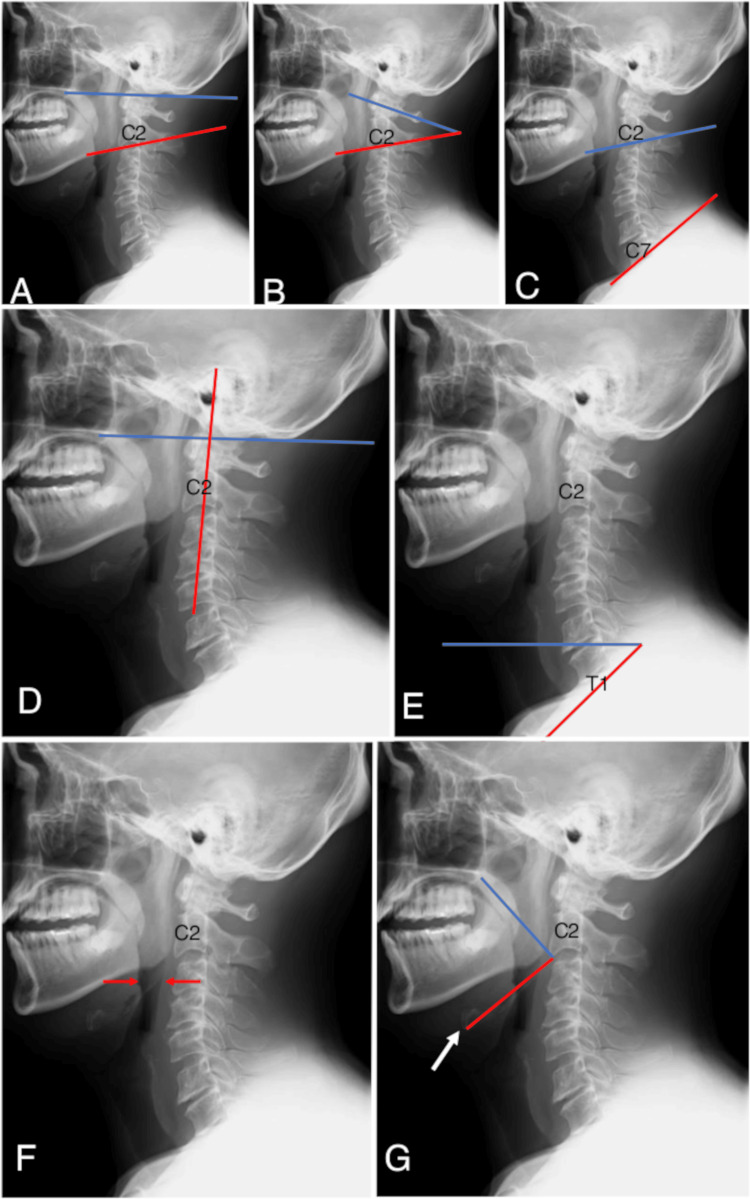
The definition of radiological parameters on X-ray images. (A) The O-C2 angle is defined by the McGregor line (blue line) and a line drawn parallel to the lower endplate of C2 (red line). (B) The C1-C2 angle is defined by a midline penetrating both the anterior arch and posterior arch of C1 (blue line) and a line parallel to the lower endplate of C2 (red line). (C) The C2-C7 angle is defined by a line parallel to the lower endplate of C2 (blue line) and a line parallel to the lower endplate of C7 (red line). (D) The pharyngeal tilt angle is defined by the McGregor line (blue line) and a line penetrating both the center of the pedicle of C2 and the center of the vertebral body at the apex of cervical sagittal curvature (red line). (E) The T1 slope is defined by the horizontal line (blue line) and a line parallel to the upper endplate of T1 (red line). (F) Pharyngeal airway space was defined as the width of the oropharyngeal cavity at the highest level of the epiglottis. (G) The hard palate-C2-hyoid bone angle was defined as the angle between a line from the posterior margin of the hard palate to the anterior margin of the lower endplate of C2 (blue line) and a line from the anterior margin of the lower endplate of C2 to the anterior margin of the hyoid bone (white arrow) (red line).

We analyzed the following pre- and postoperative (postoperative day one) angles on cervical static position X-ray images of the enrolled cases, such as the O-C2 angle, C1-C2 angle, C2-C7 angle, pharyngeal tilt angle (PTA), T1 slope, pharyngeal airway space (PAS), and hard palate-C2-hyoid bone (H2H) angle. The O-C2 angle was defined as the angle between the McGregor line and a line drawn parallel to the lower endplate of C2 (Figure 2A). The C1-C2 angle was defined as the angle between a midline penetrating both the anterior arch and posterior arch of C1 and a line parallel to the lower endplate of C2 (Figure 2B). The C2-C7 angle was defined as the angle between a line parallel to the lower endplate of C2 and a line parallel to the lower endplate of C7 (Figure 2C). The PTA was defined as the angle between the McGregor line and a line linking the center of the C2 pedicle and the center of the vertebral body at the apex of the cervical sagittal curvature (Figure 2D). T1 slope was defined as the angle between the horizontal line and a line parallel to the upper endplate of T1 (Figure 2E). PAS was defined as the width of the oropharyngeal cavity at the highest level of the epiglottis (Figure 2F). The H2H angle was defined as the angle between a line from the posterior margin of the hard palate to the anterior margin of the lower endplate of C2 and a line from the anterior margin of the lower endplate of C2 to the anterior margin of the hyoid bone. We measured this angle to indicate the relationship among the cranium, cervical vertebrae, and larynx (Figure 2G). The angles were measured by the single neurosurgeon who performed operations (M.T.).

All the data, including radiological images, were anonymized.

## Results

In total, 14 cases (male: female = 10:4) were enrolled in this study. The mean age ± standard deviation was 66.4 ± 9.7 years old (minimum age to maximum age: 31-84 years old). The underlying diseases were atlantoaxial subluxation (n=11), axis fracture (n=2), and atlas fracture (n=1). C1-C2 fixation was performed in seven cases. Occipito-C2 fixation, occipito-C3 fixation, occipito-C4 fixation, and occipito-C5 fixation were performed in one case, in three cases, in two cases, and in one case, respectively. Pedicle screws were utilized in five cases. Transarticular screws were utilized in five cases. Combined pedicle screws and transarticular screws were used in two cases. Postoperative dysphagia appeared in two cases treated with occipito-cervical fixation (cases 13 and 14) (Table 1).

**Table 1:** Summary of the enrolled cases undergoing posterior fixation surgery CLP: cervical laminoplasty, LS: lateral mass screw, O: occipital, OCF: occipito-cervical fixation PS: pedicle screw

Patients	Age (years)	Sex	Disease	Operation	Fixation levels	Devices	Postoperative dysphagia
Case 1	60	M	Atlantoaxial subluxation	C1-2 fusion	O-C3	O scerw (6), Magerl (2), C3 PS (2)	-
Case 2	74	M	Atlantoaxial subluxation	C1-2 fusion	C1-2	Magerl (2)	-
Case 3	72	M	Atlantoaxial subluxation	C1-2 fusion + CLP	C1-2	C1 LMS (2), C2 LS (2)	-
Case 4	71	F	Atlantoaxial subluxation	C1-2 fusion	C1-2	Magerl (2)	-
Case 5	66	M	Atlantoaxial subluxation	C1-2 fusion	C1-2	Magerl (2)	-
Case 6	73	M	Atlantoaxial subluxation	OCF	O-C5	O screw (6), C3 PS (2), C4 PS (2), C5 PS (2)	-
Case 7	72	F	Axis fracture	C1-2 fusion	O-C2	O screw (6), Magerl (Only left/1), C2 LS (2)	-
Case 8	84	F	Atlantoaxial subluxation	OCF	O-C4	O plate, C3 PS (2), C4 PS (2)	-
Case 9	78	M	Axis fracture	C1-2 fusion	C1-2	C1 LMS (2), C2 LS (Left), C2 PS (Right)	-
Case 10	66	M	Atlantoaxial subluxation	C1-2 fusion + CLP	C1-2	Magerl (2)	-
Case 11	73	M	Atlas fracture	OCF	O-C4	O plate, C2 LS (2), C4 PS (2)	-
Case 12	31	F	Atlantoaxial subluxation	C1-2 fusion	C1-2	Magerl (2)	-
Case 13	69	M	Atlantoaxial subluxation	OCF	O-C3	O plate, C2 LS (Left), C2 PS (Right), C3 PS (2)	+
Case 14	41	M	Atlantoaxial subluxation	OCF	O-C3	O screw (6), C2 LS (Right), C2 PS (Left), C3 PS (2)	+

Swallowing rehabilitation did not resolve postoperative dysphagia in case 13. Therefore, additional posterior re-fixation surgery was performed, and dysphagia disappeared after the second operation. In case 14, swallowing rehabilitation was effective, and no additional operation was performed. 

Radiological findings

The absolute values of chronological changes of all the radiological parameters were shown using the box-and-whisker plot.

**Figure 3:**
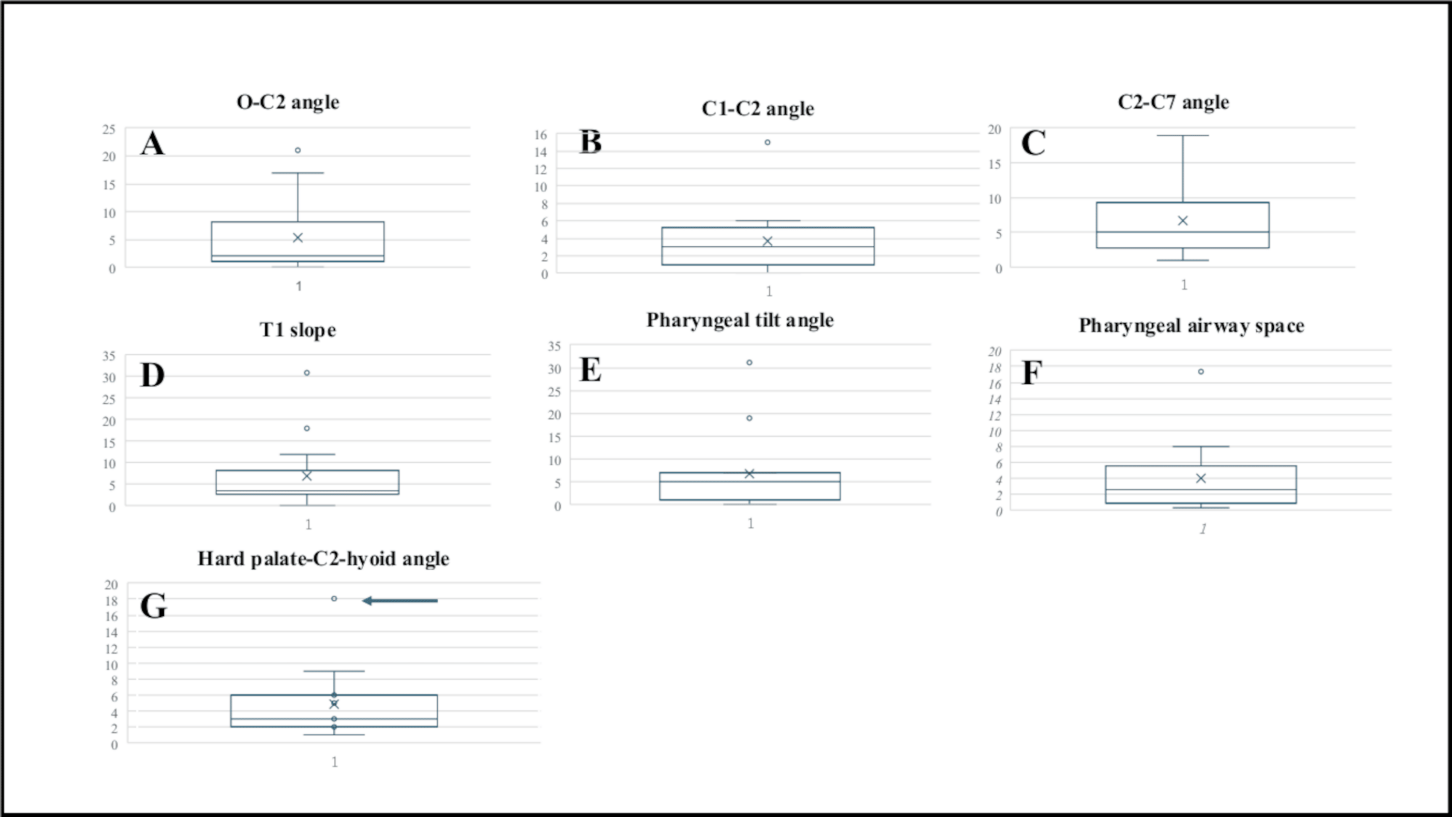
Box-and-whisker plots showing the absolute values of chronological changes in all the radiological parameters. The respective chronological changes of the radiological parameters were shown using box-and-whisker plots. The average values were shown with X-marks. The median values were shown with horizontal lines. Only in the hard palate-C2-hyoid angle was the pre- and postoperative change in one complication case plotted as an outlier (G: arrow). (A) O-C2 angle, (B) C1-C2 angle, (C) C2-C7 angle, (D) pharyngeal tilt angle, (E) T1 slope, (F) pharyngeal airway space, and (G) hard palate-C2-hyoid bone angle.

The preoperative and postoperative O-C2 angles (minimum-maximum) in the non-complication cases were 2° to 36° and 5° to 43°, respectively. The chronological change of this parameter was -8° to 21°. In the complication cases, the preoperative and postoperative O-C2 angles (minimum-maximum) were 3° to 9° and 3° to 7°, respectively. The chronological change of this parameter was -2° to 0° (Figure 3A). The preoperative and postoperative C1-C2 angles (minimum-maximum) in the non-complication cases were 11° to 43° and 13° to 48°, respectively. The chronological change of this parameter was -6° to 15°. In the complication cases, the preoperative and postoperative C1-C2 angles (minimum-maximum) were 12° to 13° and 10° to 11°, respectively. The chronological change of this parameter was -3° to -1° (Figure 3B). The preoperative and postoperative C2-C7 angles (minimum-maximum) in the non-complication cases were 7° to 27° and 2° to 36°, respectively. The chronological change of this parameter was -8° to 19°. In complicated cases, the preoperative and postoperative C2-C7 angles (minimum-maximum) were 21° to 31° and 27° to 36°, respectively. The chronological change of this parameter was 5° to 6° (Figure 3C). The preoperative and postoperative T1-slope (minimum-maximum) in the non-complication cases were 3° to 41° and 0° to 52°, respectively. The chronological change of this parameter was -31° to 18°. In the complicated cases, the preoperative and postoperative T1-slope (minimum-maximum) were 29° to 33° and 22° to 36°, respectively. The chronological change of this parameter was -7° to 3° (Figure 3D). The preoperative and postoperative PTA (minimum-maximum) in the non-complication cases were 70° to 119° and 65° to 112°, respectively. The chronological change of this parameter was -31° to 19°. In the complication cases, the preoperative and postoperative PTA (minimum-maximum) were 81° to 106° and 81° to 99°, respectively. The chronological change of this parameter was -7° to 0° (Figure 3E). The preoperative and postoperative PAS (minimum-maximum) in the non-complication cases were 7.28 cm to 24.72 cm and 6.38 cm to 30.17 cm, respectively. The chronological change of this parameter was -6.59 cm to 17.39 cm. In the complication cases, the preoperative and postoperative PAS (minimum-maximum) were 8.22 cm to 14.45 cm and 9.16 cm to 12.67 cm, respectively. The chronological change of this parameter was -5.29 cm to 4.45 cm (Figure 3F).

H2H Angles

The preoperative and postoperative H2H angles (minimum-maximum) in the non-complication cases were 62° to 119° and 60° to 122°, respectively. The chronological change of this parameter was -6° to 6°. In the complication cases, the preoperative and postoperative H2H angles (minimum-maximum) were 81° to 85° and 90° to 103°, respectively. The chronological change of this parameter was from 9° to 18°. Only in this parameter was the chronological change of one complication case plotted as an outlier (Figure 3G) (Table 2).

**Table 2:** Pre- and postoperative changes of radiological parameters on X-ray images. H2H: Hard Palate-C2-Hyoid Angle, PAS: Pharyngeal Airway Space, PTA: Pharyngeal Tilt Angle

Patients	O-C2 (°)			C1-2 (°)			C2-7 (°)			T1 slope (°)			PTA (°)			PAS (cm)			H2H (°)			Dysphagia
	Pre	Post	Δ	Pre	Post	Δ	Pre	Post	Δ	Pre	Post	Δ	Pre	Post	Δ	Pre	Post	Δ	Pre	Post	Δ	-
Case 1	23	24	1	22	22	0	10	23	13	31	0	-31	119	112	-7	18.57	18.3	-0.27	81	76	-5	-
Case 2	14	12	-2	22	19	-3	9	6	-3	20	15	-5	100	96	-4	15.77	12.87	-2.9	93	98	5	-
Case 3	2	5	3	11	13	2	27	28	1	25	26	1	75	81	6	12.13	13.06	0.93	95	92	-3	-
Case 4	14	20	6	27	29	2	26	23	-3	41	44	3	94	100	6	11.56	14.36	2.8	95	92	-3	-
Case 5	19	18	-1	28	27	-1	10	2	-8	40	36	-4	88	83	-5	11.12	11.47	0.35	90	91	1	-
Case 6	13	30	17	38	35	-3	12	17	5	35	37	2	71	72	1	7.28	6.38	-0.9	117	119	2	-
Case 7	27	25	-2	31	35	4	12	18	6	3	7	4	96	91	-5	16.22	14.32	-1.9	62	60	-2	-
Case 8	18	10	-8	26	20	-6	20	36	16	35	38	3	103	102	-1	18.55	11.96	-6.59	91	97	6	-
Case 9	20	18	-2	28	34	6	25	27	2	34	52	18	70	89	19	9.98	12.48	2.5	119	122	3	-
Case 10	22	43	21	21	36	15	10	29	19	10	22	12	96	65	-31	12.78	30.17	17.39	100	97	-3	-
Case 11	36	35	-1	31	30	-1	7	6	-1	6	6	0	99	100	1	24.72	22.33	-2.39	94	96	2	-
Case 12	23	32	9	43	48	5	7	12	5	20	17	-3	105	104	-1	7.59	15.55	7.96	94	88	-6	-
Case 13	9	7	-2	13	10	-3	21	27	6	29	22	-7	106	99	-7	8.22	12.67	4.45	85	103	18	+
Case 14	3	3	0	12	11	-1	31	36	5	33	36	3	81	81	0	14.45	9.16	-5.29	81	90	9	+

## Discussion

In this case-series study, we measured radiological parameters on X-ray images before and after OCF and upper cervical posterior fixation. Postoperative dysphagia appeared in two cases treated with OCF. Chronological changes of H2H angles in those two patients were relatively large compared to those in the other 10 cases. Because the H2H angle is composed of essential anatomical structures of swallowing, such as the palate and hyoid bone, we thought this angle could predict postoperative dysphagia after OCF and upper cervical posterior fixation.

Swallowing is a continuous movement composed of the oral, pharyngeal, and esophageal phases. During these phases, initially, food gets chewed and becomes a bolus in the oral cavity. Then, the food bolus is conveyed from the oral cavity to the pharynx as the tongue pushes the bolus dorsally. Simultaneously, the soft palate elevates and prevents the food bolus from entering the nasal cavity. In the pharyngeal phase, the cricopharyngeal muscles of the pharynx, including the superior, middle, and inferior pharyngeal constrictor muscles, begin to contract and push the food bolus toward the esophagus. To prevent food from accidentally entering the trachea, the epiglottis drops and covers the larynx [16]. A similar swallowing mechanism functions when liquid texture enters the oral cavity. Especially, laryngeal closure begins with the elevation of the hyoid bone, and this step is followed by the aperture of the pharyngoesophageal segment, closure of the vocal cord and laryngeal vestibule, and inversion of the epiglottis [17]. Thus, the palate and hyoid bone are essential structures in swallowing. However, these structures have not been reflected in radiological parameters related to postoperative dysphagia after OCF and upper cervical posterior fixation. The chronological change of the width of the oropharyngeal cavity at the highest level of the epiglottis is evaluated only in PAS, not including the palate and hyoid bone [1,18,19].

The postoperative dysphagia following OCF and upper cervical posterior fixation can result from the obstructed pharyngeal space because of the cervical flexed position and loss of the upper cervical range of motion [5,7,20]. The occipito-C1-C2 level contributes 40% of all cervical flexion and extension and 60% of cervical rotation [21]. OCF and upper cervical posterior fixation can sacrifice these motions. The following parameters were reported as risk factors for postoperative dysphagia after OCF: ΔO-C2 angle, ΔPAS, and postoperative PAS [2]. Wang et al. measured the narrowest PAS in 109 patients who underwent OCF and reported the significant decrease of this parameter in the group with postoperative dysphagia [8]. In our study, possibly because of the small sample, postoperative dysphagia occurred in a case whose PAS increased postoperatively. 

Although postoperative dysphagia was not observed in our cases treated with only upper cervical posterior fixation, the correlation between overextension or flexion reflected on O-C2 and C1-C2 angles and postoperative dysphagia after upper cervical posterior fixation was described [3,4]. The differences between preoperative and postoperative O-C2 and C1-C2 angles may also be risks of inducing dysphagia after upper cervical posterior fixation, while preoperative middle and lower cervical lordosis measured with the C2-C7 angle can be a preventive factor of this complication [4]. Kaneyama et al. assessed the relationship between PTA and postoperative dysphagia in 32 patients who underwent occipitospinal fusion. A high incidence of postoperative dysphagia after occipitospinal fusion was significantly identified in cases with PTA of less than 77°[22]. PTAs were higher than 77° in our cases with postoperative dysphagia after OCF. As this parameter was originally in patients undergoing occipitothoracic fusion, the reported results may not be simply applied to our study. However, PTA has not been often evaluated in patients after OCF or upper cervical posterior fixation. Thus, although the proposition of the H2H angle is our key message, this article can be clinically important in that PTA was evaluated in patients after OCF or upper cervical posterior fixation.

We proposed the H2H angle because any previous radiological parameters related to dysphagia after OCF and upper cervical posterior fixation did not include the palate and hyoid bone. The chronological change of H2H angles in the two cases with postoperative dysphagia after OCF was 9° and 18°, respectively. The absolute values of these numbers were relatively bigger than those in patients without postoperative dysphagia. Although the H2H angle was evaluated only in 14 patients in this study, our results might signify that the palate, C2, and hyoid bone relationship should be preserved to maintain the preoperative natural swallowing route. This hypothesis should also be tested in further research. However, our proposal seems unique because the H2H angle might be more related to the swallowing pathway than the previous radiological parameters.

Limitation

As this is a retrospective single-center study that includes a limited number of patients (approximately only 10% of our original database), the usefulness of the H2H angles should be evaluated in further studies enrolling a large number of patients undergoing OCF and upper cervical posterior fixation. The incidence of postoperative dysphagia was low because the number of enrolled patients was small. Because of these limitations, statistical analysis was not sufficient in this study. Second, treatment options performed for patients enrolled in this study were quite varied (OCF and upper cervical posterior fixations for multiple levels). Besides, patients' age and surgical instrumentation used in this study could have affected the occurrence of postoperative dysphagia. In this regard, a selective bias was not completely excluded. Thirdly, we did not examine the degree of postoperative dysphagia based on a scale (i.e., dysphagia severity scale [23,24]).To assess the degree of postoperative dysphagia, a videofluoroscopic swallowing study or flexible endoscopic evaluation of swallowing should be considered. 

However, once evidence of the H2H angles is proved, this parameter may also be predictive to avoid postoperative dysphagia following OCF and upper cervical posterior fixation.

## Conclusions

Our case-series pilot study demonstrated the potential utility of the H2H angle concerning postoperative dysphagia after OCF and upper cervical posterior fixation. This radiological parameter is unique because it reflects the anatomical relationship among the upper cervical spine, hyoid bone, and palate.

In addition to previous radiological parameters, such as PAS, O-C2, C1-C2, C2-C7, and PT angles, our pilot study seems to have clinical importance in suggesting the utility of the H2H angle, possibly related to postoperative dysphagia. However, because of the limited number of enrolled patients in this study, statistical analysis does not seem to be sufficient. Further research, including more patients after OCF and upper cervical posterior fixation, should evaluate the utility of this radiological parameter.
